# Awareness of Balance as an Intraindividual Dynamic of Objective and Subjective Experiences of Fall Risk in Daily Life

**DOI:** 10.1093/geroni/igab046.1109

**Published:** 2021-12-17

**Authors:** Shannon Meija, Tai-Te Su, Aileen Griffin, Faith Washington, Jason Fanning, Jacob Sosnoff

**Affiliations:** 1 University of Illinois, Champaign, Illinois, United States; 2 University of Illinois at Urbana-Champaign, United States; 3 University of Illinois at Urbana-Champaign, Aurora, Illinois, United States; 4 Wake Forest University, Winston Salem, North Carolina, United States; 5 School of Health Professions, University of Kansas Medical Center, Kansas City, Kansas, United States

## Abstract

Falls are life-changing events in older adulthood. With an accurate understanding of balance, older adults can adapt to age-related changes in physical ability without prematurely restricting physical activity. The Daily Balance Project examines the implications of older adults’ awareness of fall risk in daily life. For 30-consecutive days, following a fall-risk assessment, 40 older adults used a smartphone to report balance confidence and then perform four balance assessment and a 30-second sit-to-stand task to measure postural sway and fall-risk. Measures of postural sway showed greater intraindividual variability than balance confidence and fall risk. Multilevel models showed that awareness of balance fluctuated during the study and varied across individual differences in baseline fall-risk. Baseline fall risk also differentiated how balance confidence and postural sway were linked to subsequent momentary fall risk assessments. The findings are discussed within the framework of action-perspectives of adult development and awareness of aging.

